# Collision tumor of the thyroid: follicular variant of papillary carcinoma and squamous carcinoma

**DOI:** 10.1186/1477-7819-4-65

**Published:** 2006-09-19

**Authors:** Rohan R Walvekar, Subhadra V Kane, Anil K D'Cruz

**Affiliations:** 1 Department of Head and Neck Surgery, Tata Memorial Hospital, Parel, Mumbai, India; 2 Department of Pathology, Tata Memorial Hospital, Parel, Mumbai, India

## Abstract

**Background:**

Collision tumors of the thyroid gland are a rare entity. We present a case of a follicular variant of papillary carcinoma and squamous carcinoma in the thyroid. To the best of our knowledge, this is the first documentation of a collision tumor with a papillary carcinoma and a squamous carcinoma within the thyroid gland. The clinicopathological features and immunohistochemical profile are reported. The theories of origin, epidemiology and management are discussed with a literature review.

**Case presentation:**

A 65 year old woman presented with a large thyroid swelling of 10 years duration and with swellings on the back and scalp which were diagnosed to be a follicular variant of papillary thyroid carcinoma with metastasis. Clinical examination, radiology and endoscopy ruled out any other abnormality of the upper aerodigestive tract. The patient was treated surgically with a total thyroidectomy with central compartment clearance and bilateral selective neck dissections. The histopathology revealed a collision tumor with components of both a follicular variant of papillary carcinoma and a squamous carcinoma. Immunohistochemical analysis confirmed the independent origin of these two primary tumors. Adjuvant radio iodine therapy directed toward the follicular derived component of the thyroid tumor and external beam radiotherapy for the squamous component was planned.

**Conclusion:**

Collision tumors of the thyroid gland pose a diagnostic as well as therapeutic challenge. Metastasis from distant organs and contiguous primary tumors should be excluded. The origins of squamous cancer in the thyroid gland must be established to support the true evolution of a collision tumor and to plan treatment. Treatment for collision tumors depends upon the combination of primary tumors involved and each component of the combination should be treated like an independent primary. The reporting of similar cases with longer follow-up will help define the epidemiology, biology and establish standardized protocols for treatment of these extremely rare tumors.

## Background

Collision tumors within the thyroid gland are extremely rare and reported cases have mixed histologies of follicular or papillary and medullary carcinomas [1]. The co-existence of a follicular variant of a papillary carcinoma and squamous carcinoma in the thyroid gland is unique. Detailed histopathological and immunohistochemical studies solve the questions that pertain to the origins of these tumors. Collision tumors such as the one presented, especially with a component of squamous carcinoma could denote a poor prognosis. The clinicopathological features, theories of origin and management protocols of these tumors are reviewed.

## Case presentation

A 65 year old woman presented with a long standing goiter of 10 years duration and recent swellings over the back and the scalp since 3 months. The thyroid swelling was slow growing with no history of a rapid increase in the recent past. The patient's thyroid function tests (T3, T4 and TSH) were within normal limits and she had no pressure signs and symptoms. Her past history was non-contributory. A complete physical examination revealed a large, firm multinodular swelling involving the entire thyroid gland. There was no palpable cervical adenopathy and a review of all other systems was negative. Endoscopic and radiological (barium swallow) examination of the upper aerodigestive tract were normal. Her vocal cords were mobile. Examination also revealed fluctuant swellings of the left parietal and left thoracic paraspinal regions. An ultrasound of the neck revealed a 7.4 cm large heterogeneous tumor of the thyroid with bilateral lymphadenopathy of intermediate origin at levels II and III. Fine needle aspiration cytology of the thyroid gland and the scalp lesion confirmed a follicular variant of papillary carcinoma with metastatic deposits in the scalp. A biopsy of the scalp lesion was not performed in view of fine needle aspiration cytology diagnosis. A biopsy of the paraspinal mass too showed a follicular variant of papillary carcinoma. An elevated serum thyroglobulin level (> 800 ng/ml) supported the thyroid origin of the primary tumor. The chest x-ray was normal. The patient was staged as cT3 N_0 _M_1 _(Stage IVc, AJCC – TNM) [2]. The patient underwent a total thyroidectomy with central compartment clearance and bilateral selective neck dissections (levels II, III & IV). At surgery, the tumor was confirmed to arise from the gland. There were no separate lesions to suggest the presence of a thyroglossal cyst remnant or infiltration of the thyroid gland by a primary from an adjacent organ. Histopathology revealed a collision tumor with components of both a follicular variant of a papillary carcinoma and a squamous carcinoma. In view of the large tumor and dual histology, we planned for radio iodine therapy directed toward the follicular derived component of the thyroid tumor and external beam radiotherapy for the squamous component. The patient was referred for adjuvant treatment but unfortunately did not come for follow-up. At the last follow-up on the 4^th ^August 2005 the patient was free of primary tumor; three months after completion of surgical therapy. Attempts to contact the patient via all possible resources were futile.

### Surgical pathology

#### Gross examination

The total thyroidectomy specimen measured 8.5 cm × 5.5 cm × 3.0 cms, with a solid, encapsulated tumor involving the right lobe and the isthmus. On cut surface, a tan colored fleshy tumor measuring 6.0 × 5.0 × 2.5 cms was seen with a thin rim of thyroid parenchyma partially surrounding it. Juxtaposed to this tumor was another distinct gray-white tumor measuring 3.0 × 2.5 × 2.0 cms showing a central irregular cystic area. The left lobe was unremarkable. The largest node dissected was 0.8 cm in diameter and was grossly unremarkable.

### Microscopic Examination

**Figure 1:**
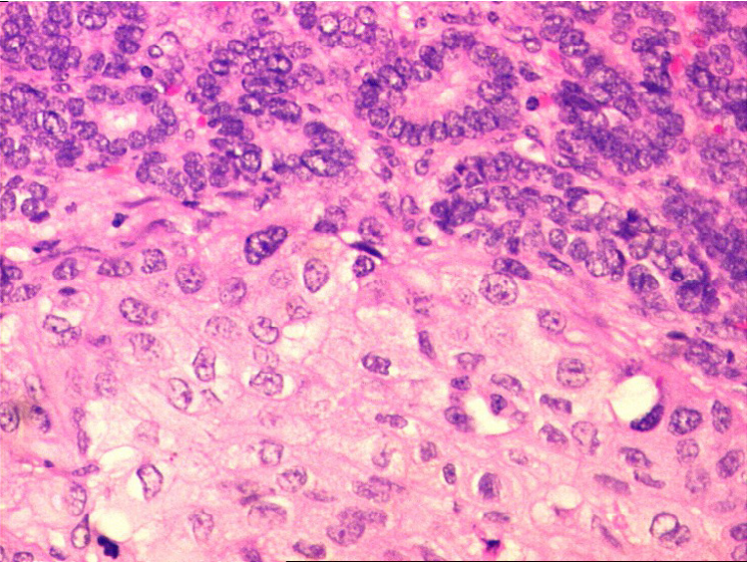
Follicular variant of papillary carcinoma of thyroid and squamous carcinoma in close juxtaposition with each other (H&E 200×).

**Figure 2:**
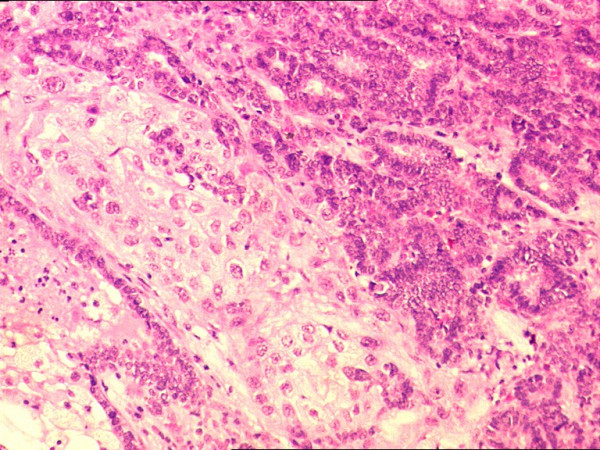
Minimal intermingling of both the tumors at the interface area without transformation (H&E 200×).

**Figure 3:**
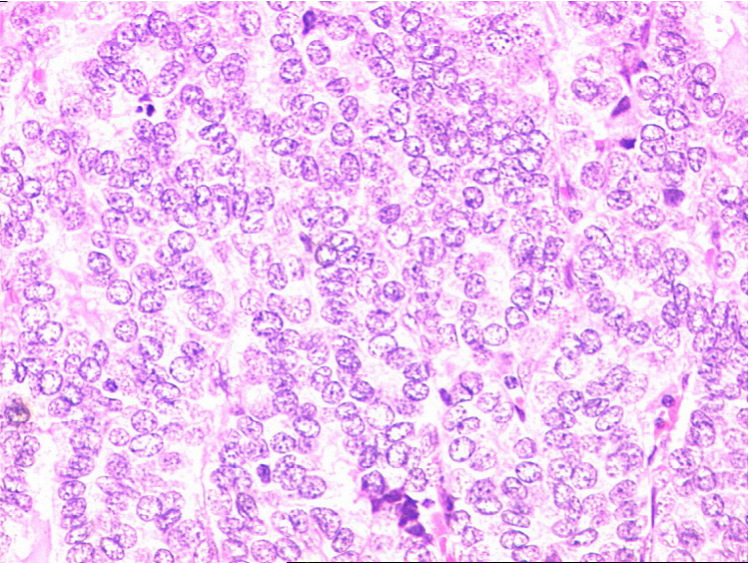
Papillary thyroid carcinoma of the thyroid showing nuclear crowding, nuclear clearing and nuclear grooves (H&E 200×).

**Figure 4:**
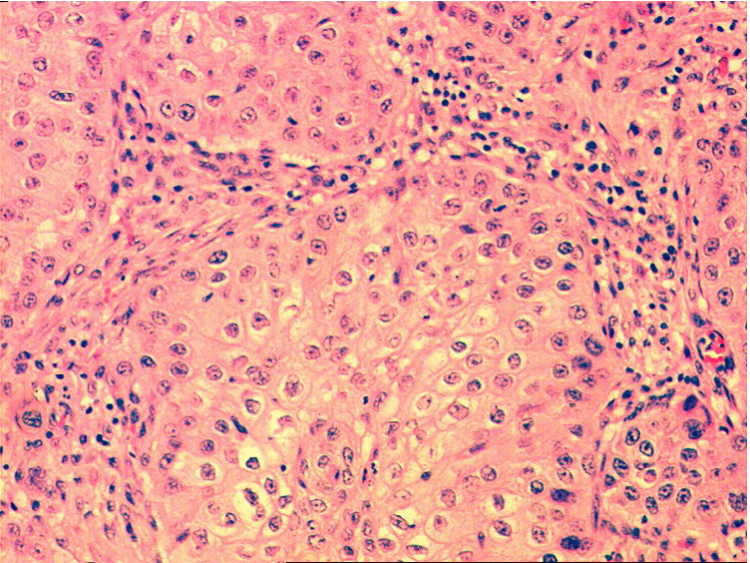
Nests of non keratinizing squamous cell carcinoma (H&E 200×).

**Figure 5:**
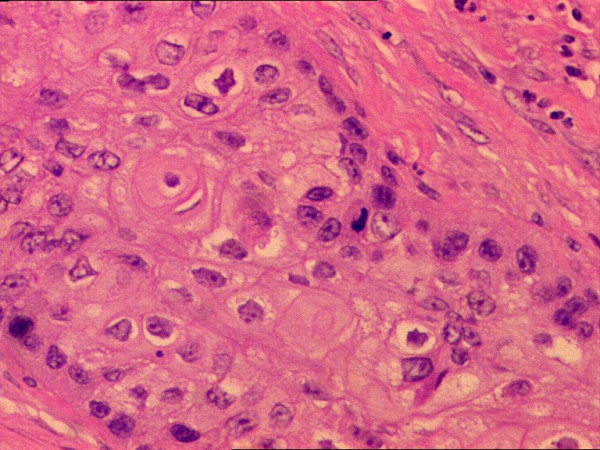
Tumor cells with eosinophilic cytoplasm forming a pearl. Note the distinct cytoplasmic borders (H&E 400×).

Multiple sections from the thyroid mass displayed an invasive follicular variant of papillary carcinoma with foci of capsular invasion. There was an obvious extrathyroidal extension of the tumor. Juxtaposed with the papillary carcinoma in close proximity was an independent primary tumour with histology of a moderately differentiated non keratinizing squamous carcinoma. The two tumours were separated by fibrous septae over a broad area. They intermingled minimally at the interface representing a true "collision tumor" (Figure 1, 2). The follicular variant of papillary carcinoma showed a uniform follicular differentiation without papillary areas or squamous morules. Classical nuclear features of papillary carcinoma namely nuclear crowding, nuclear clearing, overlapping and grooving with irregularities of the nuclear membrane were evident (Figure 3). These two tumors were distinct morphologically and were also independent without a zone of transition from papillary to squamous carcinoma. As the squamous carcinoma was non-keratinizing in nature, keratin pearls were not seen but clusters of cohesive cells with abundant eosinophilic cytoplasm and distinct cytoplasmic borders were noted (Figure 4, 5). The squamous carcinoma revealed a large area of cystic degeneration in the center and also showed muscle infiltration at the periphery. A diligent search failed to reveal remnants of thyroglossal cyst or areas of anaplastic carcinoma. There was an absence of lymphoid tissue around the component of squamous carcinoma. The uninvolved lobe showed no evidence of Hashimoto's thyroiditis.

One of the fourteen dissected nodes showed a focus of metastatic papillary carcinoma (micrometastasis) without extranodal invasion. The disease was staged histopathologically as pT3 N_1 _M_1 _(Stage IVc, AJCC – TNM) [2].

### Immunohistochemical Profile

The components of papillary carcinoma were strongly positive for thyroglobulin and TTF1 (Thyroid transcription factor) while they were negative for calcitonin. The squamous carcinoma cells were positive for cytokeratin only but negative for thyroglobulin, TTF1 and calcitonin, confirming the independent origins of the tumors.

## Discussion

The term "collision tumor" refers to coexistent but independent tumors that are histologically distinct [1]. Collision tumors can occur within the same organ or adjacent organs or in conjunction with a systemic malignancy or as a metastatic phenomenon [1]. Various mechanisms have been proposed for collision tumors. The first, is a "chance accidental meeting" of two primary tumors. Another hypothesis suggests that the presence of the first tumor alters the microenvironment facilitating the development of the second primary tumor or seeding of metastatic tumor cells. The third theory suggests a common stem cell of origin for the two tumors [1]. Multicentricity of thyroid carcinoma especially papillary carcinoma is not a rare event, occurring in a range of 18% to 22% of thyroid neoplasms. However, the presence of morphologically and histogenetically dissimilar primary neoplasia within the thyroid gland is very unusual [3]. Collision tumors of the thyroid must be differentiated from mixed and composite tumors which show parafollicular and follicular derived cellular elements. Mixed tumors have a common cell of origin; tumor cells show expression of both thyroglobulin and calcitonin. Composite tumors on the other hand, have two discrete cellular populations – thyroglobulin positive and calcitonin positive [3]. These definitions automatically rule out the presence of a mixed or composite tumor in our case which has a co-existent follicular variant of a papillary carcinoma and a squamous carcinoma.

The origin of squamous cells within the thyroid gland has many theories. These can be found as a result of persistence of thyroglossal duct or from a branchial pouch. They may also arise from a squamous metaplasia in a papillary carcinoma, anaplastic carcinoma, Hashimoto's thyroiditis or other conditions [4,5]. There was no evidence of squamous metaplasia or transformation from one tumour to the other. The histological features of both tumours were distinct and remained consistent in our examination. The de novo occurrence of primary squamous cancer from follicular cells has also been advocated. However, such primary squamous cancers of the thyroid are extremely rare and account for less than 1% of all thyroid neoplasms. Moreover the tumour cells stain positively with thyroglobulin. Lack of thyroglobulin positivity in squamous carcinoma cells ruled out the possibility of follicular epithelial origin in our case.

Primary squamous cancers of the thyroid gland are innately aggressive tumors and typically present with a high incidence of pressure symptoms (dysphagia and dyspnea), infiltration of the surrounding soft tissue and history of recent onset of the symptoms, usually within weeks or months [5]. Moreover, squamous cell carcinomas are the commonest metastatic disease in the head and neck [6]. Hence, it is important to rule out infiltration of the thyroid gland from an adjacent organ and metastasis from a distant organ before labeling a squamous cell carcinoma as a primary thyroid cancer. In our case, the squamous cancer was restricted to the thyroid gland. Absence of symptoms, a negative physical examination and a negative review of systems helped to exclude a metastatic squamous carcinoma. Although metastatic squamous cell cancers most commonly present themselves in the cervical nodes [7], a true exclusion of a metastatic disease from secondary sites would warrant an autopsy. In retrospect, a histopathological examination of the scalp lesion could have helped. Given the fact that skin metastasis especially to the scalp from a follicular variant of papillary carcinoma are extremely rare [8]; a finding of a pure thyroid metastasis would have confirmed the findings in this case and also would have excluded a possibility of a primary cutaneous squamous cell carcinoma of the scalp with metastasis to the thyroid.

Thyroglossal duct remnants, most typically thyroglossal cysts, harbor carcinoma in less than 1% of cases. 95% of these cancers are papillary carcinomas and only 5% are squamous cancers, the latter having a worse prognosis [9]. Literature has described only two cases of concurrent papillary and squamous carcinomas in the thyroglossal cyst as yet, reflecting the rarity of this histopathological combination [10]. However, localization of a carcinoma to a clearly demonstrable thyroglossal duct and a normal thyroid gland are a prerequisite to diagnose a primary thyroglossal cyst carcinoma [11]. These criteria rule out the contributions of a thyroglossal duct cyst to the development of the collision tumor in our case, as both tumors clearly arose within the thyroid gland and remnants of thyroglossal cyst were not seen either on preoperative sonography or after extensive sampling of the specimen.

Metastasis to the thyroid gland is not as unusual as previously believed. The incidence of thyroid gland involvement in autopsy studies ranges from 1.25% – 24.2%. The primary tumor can usually be identified in 95% of the cases. The presence of metastasis to thyroid indicates a disseminated disease and reflects a very poor prognosis, with average survival from diagnosis to death of 2 months. However, a previous history of a malignancy is essential to make this diagnosis [12]. Another source of a squamous cell carcinoma in the thyroid could be an invasion from a carcinoma of the larynx, tongue base or esophagus; which commonly invade the thyroid gland [13]. A normal head and neck examination, panendoscopy and barium swallow ruled out the possibility of infiltration of the thyroid gland by a primary in an adjacent organ.

These observations validate our presentation of a collision tumor in the thyroid composed of a squamous carcinoma and a follicular variant of papillary carcinoma i.e. morphologically different primary malignant tumors.

Even though metastatic disease of the thyroid have a poor prognosis in general, Shaha *et al*, in the study of 44 patients followed for an average of 20 years, have shown a long term survival of 43% in patients who had distant metastasis at presentation [14]. Our patient was offered surgery followed by radioiodine treatment. However, the presence of a squamous carcinoma infiltrating the skeletal muscles in this collision tumor prompted the addition of external beam radiotherapy because squamous cell carcinomas of the thyroid are not known to take up radio iodine [15]. It is well acknowledged that primary squamous carcinoma of the thyroid tends to have a poor prognosis and majority of the patients die within 1 year of presentation in spite of using combination therapy including surgery, radiotherapy and chemotherapy [16]. However on the other hand, Cook et al have shown that long-term survival may be possible if the disease is diagnosed early, resected completely and treated with surgery followed by radical radiotherapy [17]. In either case, we feel that there is insufficient literature to extrapolate this to predict how squamous carcinoma will behave as a component of a collision tumor. The absence of squamous carcinoma in the node metastasis and distant metastatic sites suggests an overall predominance of a differentiated thyroid cancer.

## Conclusion

Collision tumors of the thyroid are extremely rare and pose a diagnostic as well as therapeutic challenge. Metastasis from distant organs and contiguous primary tumors should be excluded. The origins of squamous cancer in the thyroid gland must be evaluated to establish the true evolution of a collision tumor and to plan treatment accordingly. Treatment guidelines are poorly defined due to the dearth of literature on this subject. Treatment for collision tumors should depend upon the combination of primary tumors involved and each component of the combination should be treated like an independent primary. In this case, the slow growing tumor, larger size and nodal metastasis suggested a predominance of the differentiated thyroid cancer. Hence surgery followed by a combination of adjuvant radioiodine and external radiotherapy was justified owing to the relatively better survival rates reported in differentiated thyroid cancer even in the presence of distant metastasis. It is vital to acknowledge the aggressive nature of primary squamous cancers of the thyroid and to be vigilant in the follow-up of these patients. A short follow-up is a limitation of this report as a longer follow-up would have provided the opportunity to study the course of the disease process. The reporting of similar cases with longer follow-up will help define the epidemiology, biology and establish standardized protocols for treatment of these extremely rare tumors.

## Competing interests

The author(s) declare that they have no competing interests.

## Authors' contributions

**RRW**: Took part in the gathering of information, literature review, retrieved articles, wrote the first draft and took part in the care of the patient. **SVK**: Took the photographs of the surgical specimen, prepared the microphotographs, added to the literature review and contributed to the manuscript content and preparation **AKD**: Critical revision and supervision.

All authors read and approved the final manuscript.

## Funding source

None
